# The implications of male human papilloma virus infection in couples seeking assisted reproduction technologies

**DOI:** 10.4274/jtgga.2017.0031

**Published:** 2018-03-01

**Authors:** Gamze Sinem Çağlar, Nicolas Garrido

**Affiliations:** 1 Department of Obstetrics and Gynecology, Ufuk University School of Medicine, Ankara, Turkey; 2 Andrology Laboratory and Sperm Bank at the Instituto Valenciano de Infertilidad, Valencia, Spain

**Keywords:** Human, papilloma virus, assisted reproduction, male infection

## Abstract

Human papilloma virus (HPV) is one of the most common viral sexually-transmitted diseases worldwide. The prevalence of HPV is higher in infertile males when compared with fertile men and ranges between 10 and 35.7% in men affected by unexplained infertility. HPV can bind to spermatozoa and can potentially be transferred to fertilized oocytes. Viral detection in blastocysts and trophoblastic cells is associated with impaired embryo development and poor pregnancy outcomes. Nevertheless, attempts to eliminate HPV-DNA from sperm samples through routine washing techniques have failed. In assisted reproduction technologies (ART), intracytoplasmic sperm injection involves no natural selection of the sperm cell, which means that these procedures have a plausible risk of injecting sperm containing HPV. The possible detrimental effects of HPV on ART in couples with infected male partners are summarized in this review.

## Introduction

Human papilloma virus (HPV) is one of the most common viral sexually-transmitted diseases in the world. The prevalence of HPV is 40% in the general population (1). HPV infection in men is considered transient and usually clears spontaneously over time. The clinical finding in men is usually penile warts on the external genitalia. At present, there is neither an effective treatment nor a control strategy for HPV infection in men. Men are generally treated through removal of the infected tissue. The presence of HPV has also been documented in the reproductive system (testis, epididymis, and ductus deferens) (2,3,4,5,6) and in semen (4,7).

The consequences of HPV in the male reproductive system have been the focus of recent research. Meanwhile, many authors reported that HPV infection was associated with reduced sperm motility and idiopathic asthenozoospermia (8,9). In addition, in infected patients, there are significantly more anti-sperm antibodies positive sperm cells (10). Male fertility impairment in HPV-DNA infection was widely reviewed by Gizzo et al. (11). Whether HPV infection in males is a casual factor for infertility still needs to be elucidated. Another debate is the possible detrimental effects of HPV on assisted reproduction technologies (ART) in couples with infected male partners, which will be summarized in this review.

## The prevalence of HPV in infertile patients

Among the sexually transmitted diseases, HPV is the most prevalent in semen (38.1%). The prevalence of HPV semen infection is 53.8% in patients with genital warts, 40.9% in males with infected partners, 10.2% in infertile patients, and 2.2% fertile controls (12). In a literature review of the last two decades, higher percentages of HPV infection were found in infertile men compared with fertile controls, and a possible correlation between HPV sperm infection and unexplained male infertility (9). Recently, the data from 1138 males showed that 6.7% of fertile males (n=523) and 17% of idiopathic infertile males were infected with HPV (n=615) (13). In a meta-analysis that evaluated HPV prevalence, 16% [95% confidence interval (CI): 10-23%] was reported from seven studies in the infertile group, whereas it was 10% (95% CI: 7-14%) in 11 reports from other populations (7). In 2015, a systematic literature review of the last two decades found that the prevalence of HPV in men ranged between 10 and 35.7% in unexplained infertility (9).

In a large study, seminal HPV was positive in 16% of fertility clinic attendees (14). In infertile patients undergoing *in vitro *fertilization (IVF)-intracytoplasmic sperm injection (ICSI) cycles, the reported prevalence of male HPV infection was 9.5% in a prospective study by Perino et al. (15), and 7.8% in the study of Schillaci et al. (8). In sperm donors, the prevalence of HPV was 16% in the study of Kaspersen et al. (16).

According to a recent epidemiologic classification, the HPV genotypes were considered low or high (oncogenic) risk (17). In most infected men, oncogenic type HPV is detected (7). Kaspersen et al. (16) reported oncogenic type HPV in 66.7% of cases, and Gimenes et al. (18) reported it in 82.7% of cases. HPV16 is the most prevalent type (23.4%), followed by HPV31, HPV51, and HPV56 (each type detected in 10.9%) and HPV42 (9.4%) (14). Infection with two or more genotypes simultaneously was observed in 5.3% of the cases in the study of Kaspersen et al. (16); 34% in the study of Foresta et al. (19), and up to 58.6% (17/29) in that of Gimenes et al. (18).

The prevalence of HPV in specific groups of patients needs to be confirmed in larger populations with an assay that is validated for use in sperm samples. Unfortunately, a ‘gold standard’ test for subclinical HPV infection for men is lacking. Unlike cervical liquid-based cytology tests, the problem of detecting HPV in semen samples is that sperm contains many inhibitors, and the currently available commercial HPV tests are not validated for use in sperm samples. Polymerase chain reaction is usually used to detect male infection in semen (12). However, HPV DNA present in semen plasma only represents part of the detectable HPV DNA. HPV has been detected in semen and in spermatozoa, particularly in the sperm head (20). HPV16 capsids can bind to live human sperm cells (21). In infected males, HPV was detected at the sperm head in 25% of the whole sperm population (22). HPV types 6, 16, 18, and 31 can bind to the sperm cell head at or near the equatorial segment (16). High-risk HPV type 16 can attach to negatively charged glycosaminoglycans on the sperm cell surface (21,23). Moreover, syndecan-1, which is localized in the equatorial segment of the sperm head, can interact with L1 capsid protein of HPV (3). The most effective method for evaluating whether the virus is located in sperm or exfoliated cells is fluorescence in situ hybridization analysis (12). In infertility, especially men undergoing ART, detection of HPV at the spermatozoa level seems to be a prerequisite to clarify outcomes.

## Sperm washing techniques and HPV

Even if little is known about the consequences of HPV infection in couples with infertility, this infection might affect the outcome and safety of ART. Nevertheless, routine sperm washing techniques do not eliminate HPV-DNA from samples (19), and in ICSI, sperm selection techniques do not guarantee non-infected sperm injection. After sperm washing centrifugation, infected samples and infected cells do not change. A minimal effect was observed in Ficoll and swim-up protocols regarding the number of infected samples (30 and 26, respectively) (19). Much better results were obtained using the modified swim-up with heparinase-III (24,25). As mentioned before, HPV can bind to negatively charged cell-surface glycosaminoglycans. Heparin is a sulfated polysaccharide that resembles GAGs in chemical structure. Heparin molecules can directly bind to papillomavirus capsids and block their association with the sperm cell surface (21). Hence, this method for washing sperm with heparinase-III for removing HPV is promising (24) but the question remains as to whether real virions can be removed or eliminated in modified swim-up technique with heparinase-III.

## HPV transmission to fertilized oocytes

HPV is able to bind to the sperm head at or near to the equatorial segment (3,10,21). At the sperm head, HPV DNA positivity is about 25% but integration into the sperm nucleus is unclear (20). E6 gene specific mRNA of HPV type 16/18 is expressed in infected sperm cells *in vitro* (26). Infected sperm are able to transfer HPV E6/E7 genes and L1 capsid protein into the oocytes (3). In *in vitro* studies, exogenous HPV-DNA, brought by sperm cells, has been observed in cumulus cells during fertilization (26,27). Considering the data above from experimental studies, the infected spermatozoa is a vector for HPV transmission into fertilized oocytes (3,28,29) and transmission of virus-destabilized genes to oocytes during fertilization is possible.

## HPV infected male and embryo development

In addition, during recent years, investigations on HPV implications in couples seeking fertility indicated poor reproductive outcomes. Numerous authors indicated reduced fertility potential in couples with HPV-infected males because the negative impact of HPV infection was observed at various steps of human embryo development (3,21,28,29).

The negative effects of HPV infection on the early development of infected embryos depend on viral genome expression at blastocysts and trophoblasts (11,29,30). The oncogenic HPV types 16 and 18 are able to inhibit embryo development only at the 2 cells stage, and there is a reduction in blastocyst formation (31). After HPV16 exposure, embryo development inhibition can be up to 30% (31). After HPV18 exposure, hatching at the blastocyst stage can be disturbed (31). The current evidence from *in vitro* studies in blastocyst stage embryonic cells also demonstrates impaired embryo development (32). When mouse blastocysts are incubated with HPV-DNA-16 fragments, DNA fragmentation and apoptosis increases (32). Studies of patients undergoing ART also support the negative effect of HPV on human embryo development. A significantly reduced blastocyst formation rate was found in infected couples when compared with non-infected couples (54% vs. 27%, p<0.05) (33). The causal effects of HPV infection start within the very early stages of embryo development. Therefore, the possible negative role of HPV infection needs to be clarified with further *in vitro* studies.

## Implantation and pregnancy rates

Regarding implantation rates, HPV infection resulted in a 37% reduction in implantation in murine embryos (34). Supporting these data, in human ICSI cycles cumulative pregnancy rates were significantly lower in infected couples when compared with non-infected couples (38% vs. 14%, p<0.05) (33). In another prospective study that investigated infertile HPV-infected couples undergoing ART, the pregnancy rate was lower in infected couples compared with non-infected couples (15). The reported pregnancy rates in HPV negative and positive men were 33.3% and 31.6%, respectively (15). In the same study, the pregnancy rates in HPV negative and positive women were 31.1% and 42.9%, respectively (15). Others also reported that cervical HPV infection in women undergoing IVF resulted in significant reduction in pregnancies when compared with HPV-negative women (23% vs. 57%) (35). In intrauterine insemination cycles, conceiving was six times less likely to occur in women with HPV infection in cervical cytology specimens compared with women without HPV infection (1.87% vs. 11.4%) (36). Data about HPV positivity and pregnancy rates are limited; however, they imply a trend towards lower pregnancy rates. However, more scientific evidence is needed to clarify this issue.

## Pregnancy outcomes

HPV is suggested as an etiologic agent of some miscarriages depending on the data, reporting that HPV is more prevalent in spontaneous abortions compared with elective pregnancy terminations (60% vs. 20%) (37). Moreover, in a recent study in infertile couples, a significantly higher abortion rate was observed in infected couples compared with non-infected couples (62.5% vs. 16.7%, p<0.05) (33). Concordantly, the rate of spontaneous abortion in women with HPV infection (n=66) was two-fold higher than that of HPV-negative women (n=900) (12% vs. 6%) (38).

The possible negative impact of HPV infection on early embryo development could result in abnormal placentation and early pregnancy loss (15,33). A few studies have shown apoptosis of embryonic cells through fragmentation in infected embryos as the probable cause of pregnancy loss (30,34,39). In 3A trophoblastic cells cultured with different types of HPV (11,16,18,31), active expression of early and late genes of HPV was observed (39). Moreover, trophoblastic cell number is reduced and trophoblastic-endometrial cell adhesion was abnormal (39,40). In trophoblastic cells transfected with HPV16, the apoptosis rate was 3- to 6-fold higher (30). In addition, HPV viral genome expression in early stages of embryo development reduces the invasiveness of trophoblastic cells (30). A progressive decrease in the invasion ability of trophoblastic cells (25-57%) from day 3 to 15 after transfection was reported (30). The down regulation of adhesion protein E-cadherin in trophoblastic cells with HPV16 viral genome expression was suggested as the possible explanation for the reduction of invasiveness of trophoblastic cells (41).

In ART cycles, the consequences of HPV infection in men might be more serious due to the plausible risk of injecting sperm containing HPV. When HPV infection is diagnosed in sperm cells of the male, the risk of pregnancy loss significantly increases (15). An abortion rate of 66% was reported in couples with HPV-infected males (15% in non-infected males) (15). According to these data, cervical HPV infection increases pregnancy loss (40% vs. 13.7%), but all pregnancies resulted in miscarriages when both partners had HPV infection (15).

On the other hand, authors investigating cervical HPV in women undergoing IVF showed no significant difference in miscarriage rates (35). The analysis of HPV-DNA in placenta from term deliveries was positive in 24%, and 17% in the placenta from spontaneous miscarriages (total n=129) (42). These rates were 15% and 24%, respectively, in the study by Conde-Ferráez et al. (43), which had 127 women in each group. Other authors (44) also confirmed that there was no causal association between HPV and pregnancy loss in a retrospective analysis of cervical HPV infection prevalence in unexplained recurrent miscarriages (n=49) and healthy controls (n=475) (26% vs. 61%, respectively).

To date, the negative impact of HPV infection demonstrated by *in vitro* studies has not been confirmed by *in vivo* studies. The adverse outcomes depending on the very early stages of embryo development cannot be demonstrated by clinical studies. Speculation will continue until a better understanding about HPV’s possible role in adverse pregnancy outcomes is obtained.

In conclusion, in couples seeking ART, despite the risk of viral transmission, HPV is not part of viral screening protocols (45). The viral screening of couples undergoing ART includes HIV, hepatitis B and hepatitis C, but not HPV (45) in the United States and also in Europe.

It remains unclear if sperm infection may have a negative impact on assisted fertilization and pregnancy outcomes. HPV is highly prevalent in the general population. Nearly 16% of the infertile population is infected (7). HPV can bind to spermatozoa and can potentially be transferred to fertilized oocytes. Viral detection in blastocyst and trophoblastic cells is associated with impaired embryo development and poor pregnancy outcomes. All the above-mentioned points raise concerns about the consequences of HPV infection in males undergoing ART. Nevertheless, the paucity of clinical data enables to draw strong conclusions.

As a first step, semen samples of risky subjects (those with genital warts and infected partners) can be screened for HPV. Secondly, caution should be recommended to eliminate HPV infection from sperm cells or reduce the infectiousness of samples before use in ART or in sperm banking. Another option for HPV-positive men might be to wait for viral clearance, which is approximately seven months for men (45,46). Moreover, detection of HPV at the spermatozoa level might be explanatory for pregnancy losses as indicated by previous studies (15,35). However, screening for HPV infection at the spermatozoa level of all couples before IVF needs an effective validated method before use in clinical practice.

Finally, large population-based studies will help to develop clinical guidelines to improve ART outcomes and ART safety in HPV-infected males. Vaccination of males might have secondary benefits because HPV is one of the most common viral sexually-transmitted diseases in the world and also a possible casual factor for infertility. However, cost-effectiveness of such a protocol needs to be confirmed before application.
